# *Drosophila* Homeodomain-Interacting Protein Kinase (Hipk) Phosphorylates the Homeodomain Proteins Homeobrain, Empty Spiracles, and Muscle Segment Homeobox

**DOI:** 10.3390/ijms20081931

**Published:** 2019-04-19

**Authors:** Eva Louise Steinmetz, Denise Nicole Dewald, Nadine Luxem, Uwe Walldorf

**Affiliations:** 1 Developmental Biology, Saarland University, Building 61, 66421 Homburg, Germany; eva.steinmetz@uks.eu; 2 Inter-Faculty Institute for Cell Biology Animal Genetics, Auf der Morgenstelle 15, 72076 Tübingen, Germany; Denise.dewald@uni-tuebingen.de; 3 MVZ centre of laboratory medicine, Viktoriastraße 39, 56068 Koblenz, Germany; n.luxem@web.de

**Keywords:** homeodomain-interacting protein kinase (Hipk), serine/threonine kinase, homeodomain, Hbn, Ems, Msh, neurogenesis

## Abstract

The *Drosophila* homeodomain-interacting protein kinase (Hipk) is the fly representative of the well-conserved group of HIPKs in vertebrates. It was initially found through its characteristic interactions with homeodomain proteins. Hipk is involved in a variety of important developmental processes, such as the development of the eye or the nervous system. In the present study, we set Hipk and the *Drosophila* homeodomain proteins Homeobrain (Hbn), Empty spiracles (Ems), and Muscle segment homeobox (Msh) in an enzyme-substrate relationship. These homeoproteins are transcription factors that function during *Drosophila* neurogenesis and are, at least in part, conserved in vertebrates. We reveal a physical interaction between Hipk and the three homeodomain proteins in vivo using bimolecular fluorescence complementation (BiFC). In the course of in vitro phosphorylation analysis and subsequent mutational analysis we mapped several Hipk phosphorylation sites of Hbn, Ems, and Msh. The phosphorylation of Hbn, Ems, and Msh may provide further insight into the function of Hipk during development of the *Drosophila* nervous system.

## 1. Introduction

Homeodomain interacting protein kinases (HIPK`s) constitute a family of serine/threonine protein kinases that are well conserved across the animal kingdom. They obtained scientific awareness for the first time when HIPK1–3 were found as interaction partners of NK homeodomain transcription factors in mammals [1]. A fourth member of this family, HIPK4 was found during the human genome project [2]. In *Drosophila,* the only orthologue of the HIPK family is DHIPK2 [3], also named Hipk [4]. Hipk is essential, as shown through loss-of-function mutations, as follows: Homozygous loss of zygotic *hipk* expression results in larval to pupal lethality, the additional loss of maternal *hipk* leads to embryonic lethality [4,5].

HIPKs/Hipk are multipurpose protein kinases that are involved in a variety of developmental and pathological processes. Their field of activity concerns the regulation of nuclear pathways involved in gene transcription, proliferation, cell survival, response to DNA damage, and differentiation (for review see [6,7,8,9]). They are usually a modulator of signal forwarding rather than one of the main components of a signaling pathway [8,10]. HIPKs/Hipk were the first cofactors of homeodomain proteins that show enzymatic activity, while the conserved DNA binding homeodomain could be shown to be essential for the interaction between homeodomain protein and the HIPK2 [1]. Therefore, homeodomain proteins are of great interest as potential substrates of Hipk.

Homeodomain proteins fulfil numerous relevant roles during vertebrate, but also during *Drosophila* development. Generally acting as DNA binding transcription factors, they initiate and regulate developmental processes like the control of the body plan and the development of the central nervous system (for review see [11]). In *Drosophila* there are 16 major classes of homeodomain proteins with the Antennapedia (Antp), Paired (Prd) and Prd-like classes being the most prominent ones [12]. The Antp class is the largest class and can be subdivided into two subclasses, the Hox-like group and the NK-like group [12]. Several homeodomain proteins comprise also other conserved domains, like the octapeptide/EH1 motif, which acts as a transcriptional repressor domain [13,14] and interacts with the co-repressor protein Groucho (Gro) [15,16].

Muscle segment homeobox (Msh) is encoded by the gene *muscle segment homeobox* (*msh*) and closely related to NK proteins, which were shown to be interaction partners of HIPKs in vertebrates [1]. The expression of *msh* starts at early embryogenesis in a bilateral series of segmentally repeated bands of dorso-lateral ectodermal cells and then changes to a ventral-lateral band from just anterior of the cephalic furrow to the posterior midgut. Later, during embryonic development, *msh* is expressed in cells at the ectoderm-mesoderm border, which corresponds to segregating neuroblasts. After this, the expression becomes more complex and alters dynamically, before the mesodermal expression declines and *msh* expression is restricted to the ventral nerve cord (VNC) and brain. The expression pattern correlates with the role of *msh* in neurogenesis and myogenesis [17]. Mutant analysis and misexpression experiments with Msh in the mesoderm demonstrated that Msh plays a pivotal role in specifying the regional identities of muscle progenitors/founders [18]. In-depth studies of nervous system specific expression also provided evidence for a function of *msh* in dorso-ventral patterning of the neuroectoderm and neuroblast specification [19,20]. In this process, the *msh* expression is controlled by the segment polarity genes *wingless* (*wg*) and *hedgehog* (*hh*), as well as components of the EGFR signaling pathway [21,22].

The homeodomain protein Empty spiracles (Ems) is encoded by the homeotic selector gene *empty spiracles* (*ems*) [23,24]. It also belongs to the NK-like proteins and contains an octapeptide/EH1 domain, like Msh. The expression of *ems* starts at the early cellular blastoderm stage in a single circumferential stripe at the anterior end of the embryo and is under control of maternal positional information, which suggested *ems* to be an anterior gap gene [23,24]. *Ems* is required for the development of structures that derive from some of the most anterior head segments, the posterior spiracles, and for the development of the tracheal system of the eighth abdominal segment of the *Drosophila* embryo [23]. Furthermore, *ems* is involved in the early development of the brain and the ventral nerve cord of *Drosophila* [23,24,25]. Later in embryonic development, *ems* is required for the formation of specific brain segments in *Drosophila*, the deutocerebrum and tritocerebrum anlagen [26]. During postembryonic development, *ems* is also involved in the development of the olfactory system. Thereby, the transcription factor is involved in the specification and pathfinding of cephalic olfactory interneurons and projection neurons, as well as in the development of larval and adult sensory organs [27,28,29].

The *homeobrain* (*hbn*) gene was originally isolated in a screen for new homeobox containing genes of *Drosophila* [30]. *Hbn* belongs to the Prd-like class of homeobox genes and is located on the right arm of the second chromosome in region 57B [30], together with the homeobox genes *orthopedia* (*otp*) and the *Drosophila Retinal homeobox* (*DRx*) [31,32]. These genes encode transcription factors that are expressed in specific regions of the *Drosophila* embryonic brain and nervous system. *Hbn* transcript expression begins at the syncytial blastoderm stage in a horseshoe like pattern in the dorsal head region. Later in embryonic development, this anterior pattern dissolves into distinct domains in the embryonic brain and appears in an additional transcript expression in each neuromere of the VNC [30].

In our search for new Hipk interaction partners, the three genes *msh*, *ems*, and *hbn* seemed to be good candidates for the following reasons. Like Hipk, they are all expressed in the embryonic nervous system and, at least *msh* and *ems*, are NK-like genes and therefore code for homeodomain proteins structurally related to known HIPK interaction partners. To test this hypothesis, we performed in vitro phosphorylation assays with GST fusion proteins of Hbn, Ems, and Msh, resulting in phosphorylation of all three proteins by Hipk.

In this work, we analyze the relationship between Hipk and the homeodomain proteins Hbn, Ems, and Msh in more detail. We demonstrate a physical in vivo interaction of these proteins with Hipk using bimolecular fluorescence complementation (BiFC) in imaginal discs. We further show that all three homeodomain proteins are substrates of Hipk in vitro and we map several of their Hipk phosphorylation sites, respectively.

## 2. Results

### 2.1. Hipk Interacts In Vivo with the Homeodomain Proteins Hbn, Ems, and Msh

**Figure 1:**
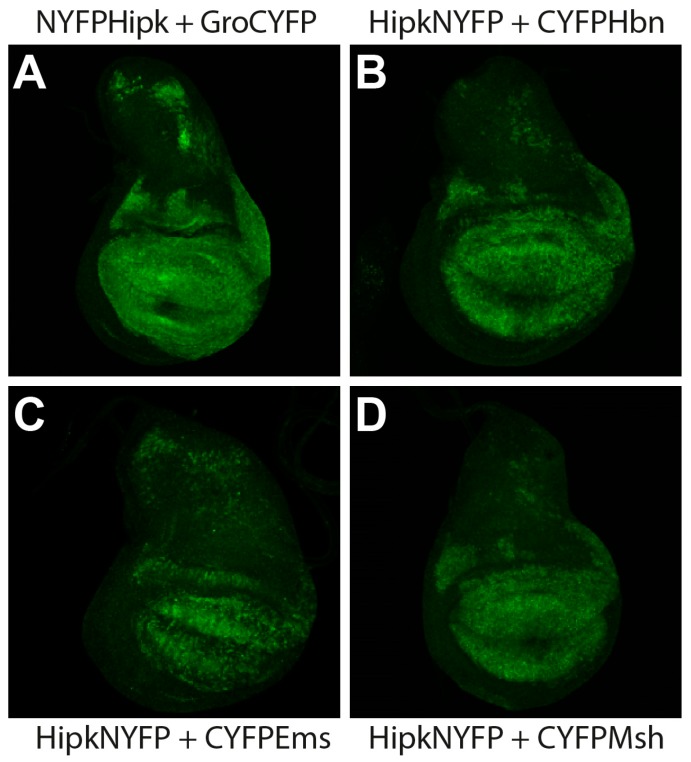
Visualization of physical interaction of Hipk with Ems, Msh, and Hbn, respectively, in vivo through bimolecular fluorescence complementation (BiFC) in *Drosophila* third larval instar wing imaginal discs. Anterior is to the left. MS1096-Gal4 was used to ectopically express UAS-BiFC-constructs. All discs were stained with anti-GFP antibody (green) to enhance the BiFC signal. (**A**) Groucho (Gro), as a confirmed substrate of Hipk, served as positive control. Co-Expression of UASNYFP*hipk* and UAS*gro*CYFP leads to physical interaction of both proteins (NYFPHipk + GroCYFP) and therefore reconstitution of YFP. (**B**) Co-expression of UAS*hipk*NYFP and UASCYFP*hbn* leads to reconstitution of YFP (HipkNYFP + CYFPHbn). (**C**) Co-Expression of UAS*hipk*NYFP and UASCYFP*ems* allows physical interaction of both proteins (HipkNYFP and CYFPEms) and therefore reconstitution of YFP. (**D**) Co-expression of UAS*hipk*NYFP and UASCYFP*msh* leads to reconstitution of YFP (HipkNYFP + CYFPMsh). For controls and for MS1096 > UAS-enhanced GFP displaying expression pattern of MS1096 driver, see Figure S1.

Hipk is expressed in the embryonic and larval central nervous system, as well as in larval structures such as wing and eye imaginal discs [5,33,34]. Hbn, Ems, and Msh are also expressed in the embryonic and larval central nervous system [17,23,24,30]. To detect potential in vivo protein interactions between Hipk and Hbn, Ems, and Msh, respectively, we used the well-established method of bimolecular fluorescence complementation (BiFC) [35]. Transgenic BiFC strains for Hbn, Ems, and Msh were generated with a C-terminal fusion of the respective full-length protein to the C-terminal nonfluorescent fragment of YFP (CYFP) under the control of upstream activation sequences (UAS). Transgenic BiFC strains for Hipk were used in both orientations to avoid steric hindrance that may lead to reduced interaction behaviour, N-terminally and C-terminally fused to the N-terminal nonfluorescent fragment of YFP (NYFP) [34]. Gro, a well-described substrate of Hipk [3,33], has been established as a systemic positive control specifically for BiFC studies on Hipk interactions and served as methodical control [34]. Gro was used N-terminally fused to CYFP, as described previously [34]. To induce targeted expression of the BiFC constructs in a wing-specific pattern, we used the Gal4 driver line MS1096-Gal4. The coexpression of UAShipkNYFP with either UASCYFP*hbn*, UASCYFP*ems*, or UASCYFP*msh* in third larval instar wing discs revealed a physical interaction of HipkNYFP with CYFPHbn, CYFPEms, and CYFPMsh, as detected by a fluorescence signal in a broad expression pattern throughout the wing pouch (Figure 1). However, when UASNYFP*hipk* was coexpressed with either UASCYFP*hbn*, UASCYFP*ems*, or UASCYFP*msh* under the control of the same driver, no fluorescence signal was detected [36]. False positive signals owing to the detection of single YFP fragments were excluded by crossing the MS1096-Gal4 driver with single UAS-BiFC lines (UASNYFP*hipk*, UAS*hipk*NYFP, UAS*gro*CYFP, UASCYFP*hbn*, UASCYFP*ems*, and UASCYFP*msh*) as controls (Figure S1).

### 2.2. Hipk Phosphorylates Hbn, Ems, and Msh

**Figure 2:**
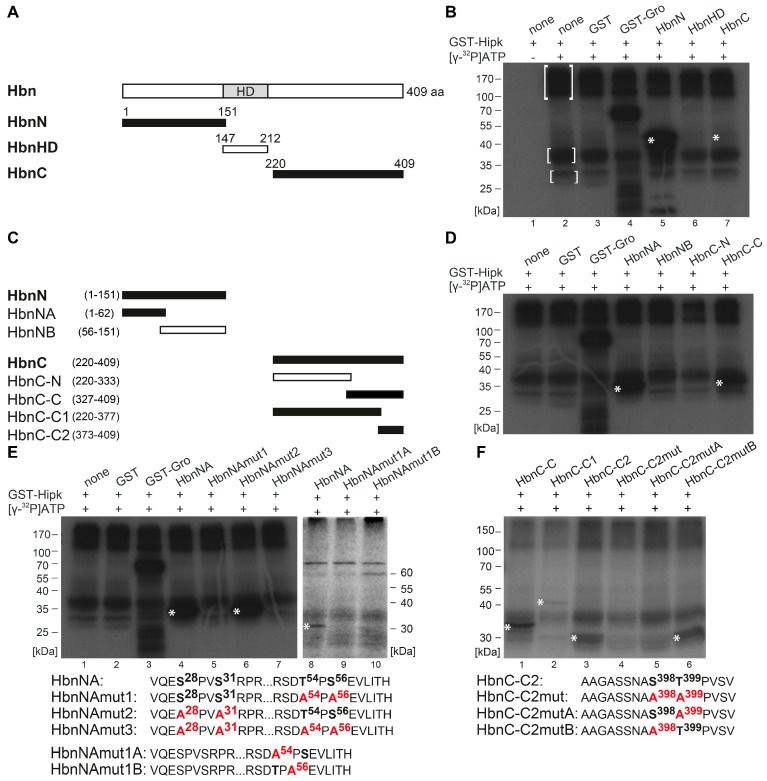
Hipk phosphorylates Homeobrain (Hbn). (**A**) Schematic view of the full-length protein Homeobrain (Hbn) with 409 amino acids (aa) and Homeodomain (HD), as well as the corresponding amino (N)-, homeodomain (HD)-, and carboxy (C)-terminal constructs that were purified and analyzed by phosphorylation assay. Numbers indicate terminal amino acid positions of the constructs. Phosphorylated constructs are represented in black and non-phosphorylated constructs are in white. (**B**) Autoradiograph after in vitro phosphorylation of purified proteins HbnN, HbnHD, and HbnC. Each protein contains a Glutathione S-transferase (GST)-tag and was incubated with full length GST-Hipk and radioactively labeled adenosine triphosphate ([γ-^32^P]ATP). Glutathione S-transferase protein (GST) served as negative control and GST-Groucho (GST-Gro), a confirmed substrate of Hipk, as positive control. There is autophosphorylation of Hipk detectable (see marked within white brackets), which is to subtract from substrate phosphorylation in following lanes. Signals from phosphorylated proteins are marked with white asterisks. Loading control is shown in Figure S2. (**C**) Schematic view of the different truncated versions of HbnN and HbnC, which were generated to locate phosphorylated regions of Hbn. (**D**) Autoradiograph after in vitro phosphorylation of purified proteins HbnNA, HbnNB, HbnC-N, and HbnC-C. (**E**,**F**) Autoradiographs after in vitro phosphorylation of purified proteins HbnNA, HbnNAmut1, HbnNAmut2, HbnNAmut3, HbnNAmut1A, and HbnNAmut1B (**E**), as well as HbnC-C, HbnC-C1, HbnC-C2, HbnC-C2mut, HbnC-C2mutA, and HbnC-C2mutB (**F**) with the concerned amino acid sequences below, respectively. Potentially phosphorylated amino acid residues that were affected by the mutational analysis are accentuated in bold.

**Figure 3:**
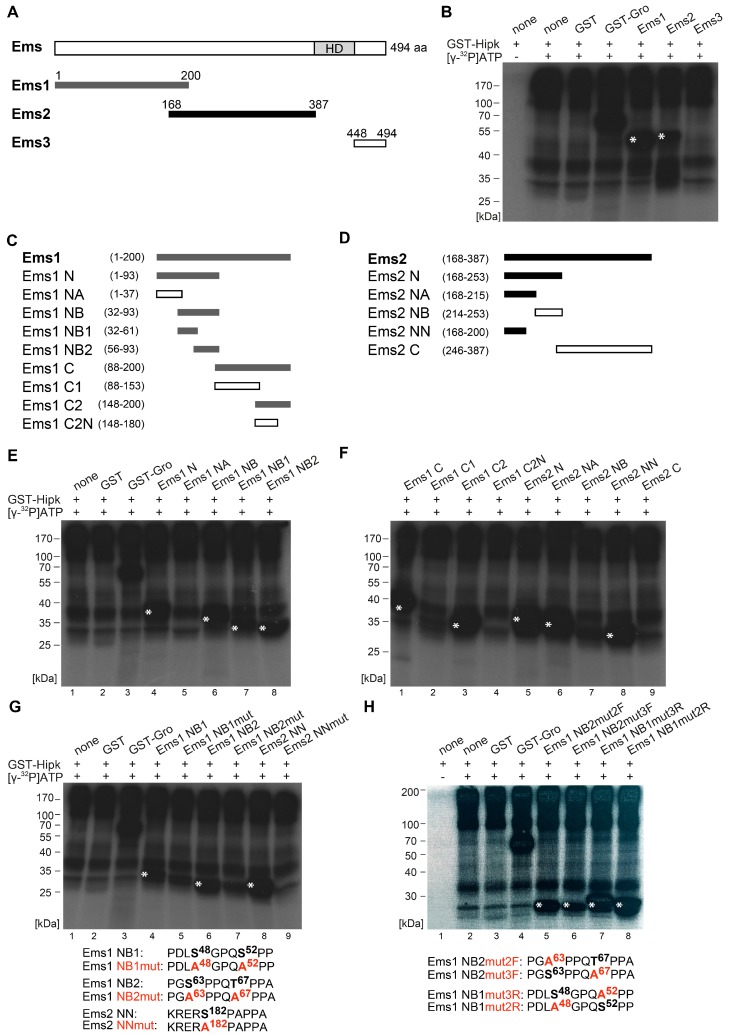
Hipk phosphorylates Empty spiracles (Ems). (**A**) Schematic view of the full-length protein Empty spiracles (Ems) with 494 amino acids (aa), Homeodomain (HD), and the corresponding amino terminal Ems1, Ems2, and carboxy terminal Ems3 constructs that were purified and analyzed by phosphorylation assay. Numbers indicate terminal amino acid positions of the constructs. Phosphorylated constructs are represented in dark grey/black and not phosphorylated constructs are in white. (**B**) Autoradiograph after in vitro phosphorylation of purified proteins Ems1, Ems2, and Ems3. Each protein contains a Glutathione S-transferase (GST)-tag and was incubated with full length GST-Hipk and radioactively labeled adenosine triphosphate ([γ-^32^P]ATP). Glutathione S-transferase protein (GST) served as negative control and GST-Groucho (GST-Gro), a confirmed substrate of Hipk, as positive control. There is autophosphorylation of Hipk detectable, which is to subtract from substrate phosphorylation in following lanes. Signals from phosphorylated proteins are marked with white asterisks. Loading control is shown in Figure S3. (**C**,**D**) Schematic view of the different truncated versions of Ems1 (**C**) and Ems2 (**D**), which were generated to locate phosphorylated regions of Ems (**E**,**F**). Autoradiographs after in vitro phosphorylation of purified proteins Ems1 N, Ems1 NA, Ems1 NB, Ems1 NB1, and Ems1 NB2 (E) and autoradiographs after in vitro phosphorylation of purified proteins Ems1 C, Ems1 C1, Ems1 C2, Ems1 C2N, Ems2 N, Ems2 NA, Ems2 NB, Ems2 NN, and Ems2 C (F). (**G**,**H**) Autoradiographs after in vitro phosphorylation of purified proteins Ems1 NB1, Ems1 NB1mut, Ems1 NB2, Ems1 NB2mut, Ems2 NN, and Ems2 NNmut (**G**) and Ems1 NB2mut2F, Ems1 NB2mut3F, Ems1 NB1mut3R, and Ems1 NB1 mut2R (**H**) with the concerned amino acid sequences below. Potentially phosphorylated amino acid residues that were affected by the mutational analysis are accentuated in bold.

**Figure 4:**
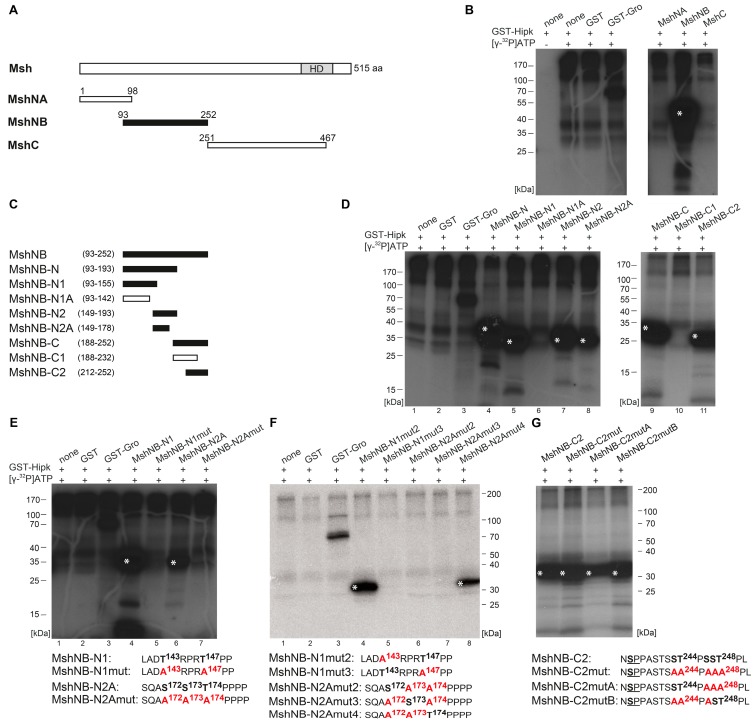
Hipk phosphorylates Muscle segment homeobox (Msh). (**A**) Schematic view of the full-length protein Muscle segment homeobox (Msh) with 515 amino acids (aa), Homeodomain (HD), and the corresponding amino terminal MshNA, MshNB, and carboxy terminal MshC constructs that were purified and analyzed by phosphorylation assay. Numbers indicate terminal amino acid positions of the constructs. Phosphorylated constructs are represented in black and not phosphorylated constructs are in white. (**B**) Autoradiograph after in vitro phosphorylation of purified proteins MshNA, MshNB, and MshC. Each protein contains a Glutathione S-transferase (GST)-tag and was incubated with full length GST-Hipk and radioactively labeled adenosine triphosphate ([γ-^32^P]ATP). Glutathione S-transferase protein (GST) served as negative control and GST-Groucho (GST-Gro), a confirmed substrate of Hipk, as positive control. There is autophosphorylation of Hipk detectable, which is to subtract from substrate phosphorylation in following lanes. Signals from phosphorylated proteins are marked with white asterisks. Loading control is shown in Figure S4. (**C**) Schematic view of the different truncated versions of Msh, which were generated to locate phosphorylated regions of Msh. (**D**) Autoradiographs after in vitro phosphorylation of purified proteins MshNB-N, MshNB-N1, MshNB-N1A, MshNB-N2, MshNB-N2A, MshNB-C, MshNB-C1, and MshNB-C2. (**E**–**G**) Autoradiographs after in vitro phosphorylation of purified proteins MshNB-N1, MshNB-N1mut, MshNB-N2A, and MshNB-N2Amut (**E**) as well as MshNB-N1mut2, MshNB-N1mut3, MshNB-N2Amut2, MshNB-N2Amut3, and MshNB-N2Amut4 (**F**) and MshNB-C2, MshNB-C2mut, MshNB-C2mutA, and MshNB-C2mutB (**G**) with the concerned amino acid sequences below. Potentially phosphorylated amino acid residues that were affected by the mutational analysis are accentuated in bold.

As a Hipk-Hbn, -Ems, and -Msh interaction could be shown in vivo, respectively, our next intention was to identify potential Hipk phosphorylation sites of these proteins. As described previously, Hipk is a proline-guided serine/threonine kinase with phosphorylation target sites within a cluster of two or three adjacent residues around a proline [37]. In the amino acid sequences of Hbn, Ems, and Msh we evaluated several potential phosphorylation clusters with the help of our preliminary Hipk-consensus motif [34,38]. Owing to the distribution of these clusters, to simplify handling and, at the same time, get a first rough mapping, we initially divided the protein-coding region of Hbn, Ems, and Msh into smaller sections (schematic overview in Figure 2, Figure 3, and Figure 4), respectively. We avoided interruption of known protein domains, potential phosphorylation clusters matching the consensus, and created overlaps at the section boundaries to prevent false negative results through site disruption.

To identify and locate regions of Hbn that are a target of Hipk phosphorylation, we performed in vitro phosphorylation analysis. Different sections were amplified and cloned for expression of recombinant protein fragments. The resulting GST fusion proteins were affinity-purified, subjected to an in vitro kinase assay with recombinant GST-Hipk and radiolabeled [γ-^32^P] ATP. A rough mapping was done with the GST fusion proteins HbnN (1–151 aa), HbnHD (147–212 aa), and HbnC (220–409 aa) (Figure 2A,B; Figure S2). While there wasn’t any phosphorylation detected within HbnHD, the N-terminal part of Hbn showed a very strong phosphorylation signal. In contrast, the phosphorylation signal of HbnC appeared very weak (Figure 2B), but was confirmed through signals in the following phosphorylation assays. To get a more accurate mapping, we analyzed both the N-terminal and the C-terminal region, with truncated subconstructs of the respective fusion proteins, and subdivided HbnN into HbnNA (1–62 aa) and HbnNB (56–151 aa), as well as HbnC into HbnC-N (220–333) and -C (327–409) (Figure 2C,D). On the basis of the truncated subconstructs we were able to locate phosphorylated regions to HbnNA and HbnC-C. The subdivision of HbnC-C (HbnC-C1 (220–377) and HbnC-C2 (373–409)) showed that Hipk phosphorylation occurs in two distinct regions. HbnC-C1 was phosphorylated, but very weakly, while HbnC-C2 showed a strong phosphorylation signal (Figure 2C,F).

We narrowed the individual regions down to single amino acid residues (serine or threonine) phosphorylated by Hipk using a mutational analysis of the previously identified subconstructs. Hereby, we introduced single amino acid exchanges from serine or threonine to alanine to find out and test potential phosphorylation sites. First, we always generated a completely mutated construct in which all the potential phosphorylation sites were exchanged to alanine (amino acid sequence of HbnNAmut3, Figure 2E; HbnC-C2mut, Figure 2F), then we generated constructs with single potential phosphorylation cluster mutated (amino acid sequence HbnNAmut1, -mut2, HbnNAmut1A and -mut1B, Figure 2E; HbnC-C2mutA and -mutB, Figure 2F). Mutational analysis of the N-terminal region of Hbn revealed that the phosphorylation cluster around Pro55 (with adjacent Thr54 and Ser56) is phosphorylated by Hipk in vitro, while the potential target cluster around Pro29 (with Ser28 and Ser31) is not (autoradiograph Figure 2E, lanes 4–7). Mutation of the single potential phosphorylation sites Thr54 and/or Ser56 revealed that if either site is destructed, the second site doesn`t get phosphorylated anymore (autoradiograph Figure 2E, lanes 8–10). Additionally, the mutation of both potential phosphorylation sites of HbnC-C2 led to the abolishment of the respective phosphorylation signal (autoradiograph Figure 2F, lane 4). Therefore, we tested single mutated phosphorylation sites in HbnC-C2mutA and -C2mutB (autoradiograph Figure 2F, lanes 5–6). The results show that Thr399, but not Ser398, is a target of Hipk in vitro. In summary, Hbn protein contains three Hipk-phosphorylation sites, Thr54, Ser56, and Thr399.

In Ems, the first sections comprise the N-terminal part Ems1 (aa 1–200), the central part Ems2 (aa 168–387), and the C-terminal part Ems3 (aa 448–494). All sections were amplified, cloned for expression of recombinant GST fusion protein fragments, and analyzed, as done for Hbn. Hipk clearly phosphorylated Ems1 and Ems2, but not Ems3 (Figure 3A,B). Therefore, Ems was validated as a substrate of Hipk in vitro, with phosphorylation in at least two regions. To obtain a more accurate mapping, we narrowed down the regions by analyzing shorter subconstructs of Ems1 and Ems2 (for a schematic view, see Figure 3C,D). A first subdivision of Ems1 showed phosphorylation in the N-terminal (Ems1 N, Figure 3E, lane 4) and the C-terminal (Ems1 C, Figure 3F, lane 1) region. Within the N-terminal subconstruct (Ems1 N), the phosphorylation could be located to the construct Ems1 NB (Figure 3E, lane 6), and finally to Ems1 NB1 and Ems1 NB2 (Figure 3E, lanes 7 and 8). The phosphorylation of Ems1 C could be assigned to the overlapping region of Ems1 and Ems2, as Ems1 C2 was phosphorylated, but Ems1 C2N was not (Figure 3F, lanes 2 and 3). Further mapping of this region was done through the analysis of the subconstructs of Ems2 (schematic view in Figure 3D). Ems2 was phosphorylated only in the N-terminal region (Ems2 N, Ems2NA, and Ems2NN, Figure 3F, lanes 5, 6, and 8), but not in the C-terminal region (Ems2NB and Ems2 C, Figure 3F, lanes 7 and 9). Through the analysis of further subconstructs, it was possible to identify a total of three phosphorylated regions within the Ems sequence. We narrowed the individual clusters down to single amino acid residues using a mutational analysis, as done for Hbn. First, we generated a completely mutated construct in which all the potential phosphorylation sites were mutated to alanine (see the following amino acid sequences in Figure 3G: Ems1 NB1mut, Ems1 NB2mut, and Ems2 NNmut). The ensuing diminishment of the phosphorylation signal confirmed the sequences as Hipk target sites (Figure 3G, lanes 5, 7, and 9). However, we could not rule out the possibility that there is another phosphorylation site, because the phosphorylation signal was not totally lost. We then generated constructs with single potential phosphorylation sites mutated (see the following amino acid sequences in Figure 3H: Ems1 NB2mut2F, Ems1 NB2mut3F, Ems1 NB1mut3R, and Ems1 NB1mut2R) to check each single putative site within a cluster. All of the potential target sites were phosphorylated (Figure 3H, lanes 5–8). Especially, Thr67 appears to be a Hipk target, as the phosphorylation signal of EmsNB2 decreased clearly after mutation to alanine (Figure 3H, lane 6). As result from the mutational analysis we could show that Ems is phosphorylated by Hipk at Ser48, Ser52, Ser63, Thr67, and Ser182.

The protein Msh was subdivided into MshNA (aa 1–98), MshNB (aa 93–252), and MshC (aa 251–467) to get a rough mapping of Hipk phosphorylation sites (schematic view in Figure 4A). MshNA and MshC were not phosphorylated, while MshNB was (Figure 4B). We then narrowed down the regions based on different successively truncated subconstructs of MshNB (schematic view Figure 4C). First, analysis of the subconstructs MshNB-N and MshNB-C (Figure 4D, lanes 4 and 9) revealed at least two regions that are phosphorylated, which were expanded to three with the help of subordinated constructs MshNB-N1, MshNB-N2, MshNB-C1, and MshNB-C2 (Figure 4D, lanes 5, 7, 10, and 11). Within the N-terminal region, the mapping was further improved by the analysis of MshNB-N1A and MshNB-N2A (Figure 4D, lanes 6 and 8). Taken together, Hipk-phosphorylation of Msh was related to the regions of MshNB-N1, MshNB-N2A, and MshNB-C2. The C-terminal region of MshNB-N2 was excluded because it contains no proline-guided serine or threonine. The C-terminal region of MshNB-N1 was identified indirectly, because MshNB-N1 was phosphorylated while the truncated version, MshNB-N1A, was not (Figure 4D, lane 5 and 6). After having located phosphorylation of Msh to the three subconstructs MshNB-N1, MshNB-N2A, and MshNB-C2, the single amino acid residues phosphorylated by Hipk were mapped using in vitro mutational analysis as done for Hbn and Ems. First, we generated completely mutated constructs in which all potential phosphorylation sites were substituted by alanine (see the following amino acid sequences in Figure 4E: MshNB-N1mut and, MshNB-N2Amut). The ensuing loss of phosphorylation signal confirmed the sequences to be targets of Hipk-mediated phosphorylation (Figure 4E, lanes 5 and 7). After this, we generated constructs with two simultaneously mutated phosphorylation sites and one site remaining intact (see the following amino acid sequences in Figure 4F: MshNB-N1mut2, MshNB-N1mut3, MshNB-N2Amut2, -mut3, and -mut4) to check each single putative target site. Only MshNB-N1mut2 and MshNB-N2Amut4 were phosphorylated by Hipk (Figure 4F, lanes 4 and 8), so we could show that Msh is phosphorylated by Hipk at Thr147 and Thr174. In the case of MshNB-C2, the mutational analysis was more complicated as the region contains a large number of potential phosphorylation sites. The potential phosphorylation clusters around Pro245 and Pro249 were analyzed by mutation of the respective adjacent serine and/or threonine residues to alanines (see amino acid sequences in Figure 4G). All mutated derivatives of MshNB-C2 were still phosphorylated. The remaining phosphorylation signal points to another phosphorylation site, which was not exclusively tested in this analysis. We propose that the remaining phosphorylation signal is due to Ser236 (next to Pro237; see underlined in amino acid sequence of MshNB-C2 in Figure 4G), which is the only remaining proline-guided phosphorylatable amino acid in this construct.

## 3. Discussion

**Figure 5:**
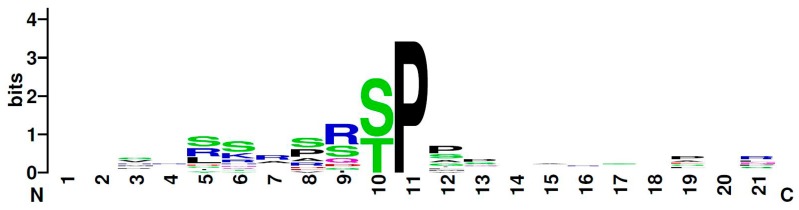
Graphical representation (logo) of the amino acid sequence from Drosophila Hipk substrates. The total height indicates the degree of sequence conservation at the corresponding position. The height of individual letters shows the relative frequency of an amino acid at this position. The Hipk consensus, according to the known Hipk substrates in *Drosophila* (Twin of eyeless, Eyeless, Groucho, Nito, Homeobrain, Empty spiracles, Muscle segment homeobox, Yorkie). The protein sequences of the substrates were aligned on the kinase-guiding proline. The graphic was created online at www.weglogo.berkeley [40].

In this study, we proved a physical in vivo interaction between the homeodomain interacting protein kinase Hipk and each of the homeodomain transcription factors Hbn, Ems, and Msh, via bimolecular fluorescence complementation assay. Subsequently, we identified all three factors as direct substrates of Hipk and mapped their phosphorylated residues. Hbn was phosphorylated in three positions (Thr54, Ser56, and Thr399) and Ems in five (Ser48, Ser52, Ser63, Thr67, and Ser182). For Msh, two positions were clearly mapped (Thr147 and Thr174), while one (Ser236) was indirectly deduced from the available data. All these phosphorylation sites are present in all *Drosophilidae* with Hbn Thr399, Ems Ser182, and Msh Thr147 offering the best sequence conservation concerning neighboring amino acids. Ems Ser182 also appeared as a phosphorylation site in a large-scale *Drosophila* phosphoproteome screen [39]. The newly identified Hipk phosphorylation sites in Hbn, Ems, and Msh also match the previously developed consensus S/X-R/X-S/T-P-S/X [34,38]. Considering those sequences, the Hipk consensus is now adapted to S/P-R/X-S/T-P-P/S (Figure 5, [40]).

The first members of the HIPK family were originally found during a yeast two-hybrid screen for interaction partners of the homeodomain transcription factor NKx-1.2 (HIPK1–3; [1]) and by sequence similarities within the human genome (HIPK4; [2]). Best analysed, and therefore referred to, as the prototype of this kinase family is HIPK2 [6,8,9,41], which is also the orthologue of the *Drosophila* Hipk, also named DHIPK2 or HIPK [3,4].

Vertebrate HIPK2 mRNA expression was detected in mouse embryonic midbrain/hindbrain regions, in developing sensory ganglia, in the developing spinal cord, as well as in different non-neural tissues, such as liver, heart, and kidney [42]. In *Drosophila*, *hipk* shows a broad mRNA expression pattern in the embryonic brain and the ventral nerve cord, emerging from a basic ubiquitous occurrence. Looking at larval brains, *hipk* mRNA appeared considerably enriched in the hemispheres [34]. Given the similar expression patterns in the respective embryonic central nervous systems, Hipk might be involved in similar processes during development of the nervous system as its orthologue HIPK2.

The homeodomain transcription factor Hbn is, like Hipk, also expressed in the nervous system. Loss of function alleles of *hbn* are embryonic lethal and show strong defects in embryonic brain development, especially a loss of the preoral brain commissure [43]. The preoral brain commissure is a structure functionally related to the human corpus callosum, which connects the two brain hemispheres. For the development of this structure, a midline patterning process, involving the specification of commissural neurons as well as axon guidance across the midline to the contralateral side [44], is necessary. If this process is disturbed, an agenesis of the corpus callosum is observed with the failure of large bundles of fibers connecting the brain hemispheres [44]. One potential cause for agenesis appeared in the context of lissencephaly form XLAG (X-linked lissencephaly with abnormal genitalia) in terms of mutations in the gene Aristaless-related homeobox (ARX). This gene is regarded as a functional homologue of *hbn* because of structural and particularly phenotypic similarities [43,45,46]. According to datasets of the online database Harmonizome, HIPK2 is shown to be expressed in prenatal human corpus callosum [47]. Although the homeodomain transcription factor ARX is no direct structural homologue of Hbn, some functional pathways, such as the general regulation by phosphorylation, might be conserved. The transcriptional activity of ARX is shown to be affected through phosphorylation by protein kinase C alpha (PRKCA) at several sites, as follows: Ser37, Ser67, and Ser174 [48]. Since several Hipk/HIPK2 substrates are additionally phosphorylated by other kinases, the opposite is also conceivable. Indeed, the phosphorylation site Ser37 (including the surrounding amino acids) fits to our Hipk consensus motif, S/P-R/X-S/T-P-P/S, and therefore looks like a putative Hipk phosphorylation site in ARX. So, it would be interesting to analyze ARX as a potential substrate of HIPK2. Altogether, it might be worth to investigate the role of HIPK2 in corpus callosum formation in general and a possible interaction between ARX and HIPK2 in particular.

With Ems, another factor involved in *Drosophila* brain development was identified as a substrate of Hipk. During post-embryonic development Ems shows a strong involvement in the formation of the olfactory system via specification and pathfinding of olfactory interneurons and projection neurons. Furthermore, the transcription factor is involved in the development of larval and adult sensory organs [27,28,29]. The mouse homologues of Ems, Emx1, and Emx2, are expressed in specific domains of the embryonic cerebral cortex [49,50]. *Emx1* mutant mice are viable and show a disruption of the corpus callosum [51], whereas *Emx2* mutant animals show more severe phenotypes like changes in the architecture of the cerebral cortex, in particular a reduction of the olfactory bulbs, an altered patterning of the hippocampus, the absence of the dentate gyrus, and they die shortly after birth [52,53,54]. It was also shown that *Emx1* and *Emx2* cooperate in the regulation of cortical size, lamination, and neuronal differentiation [55]. In vertebrates, HIPK2 is present in the developing sensory ganglia, including the trigeminal, vestibulocochlear, nodose-petrosal, and the dorsal root ganglia [42]. In the mouse sensory neurons, HIPK2 interacts with POU homeodomain transcription factor Brn3a and suppresses the expression of pro survival genes [42].

Taken together, both factors, Ems/Emx1/Emx2 and Hipk/HIPK2 are involved in sensory organ development. In *Drosophila*, we could set Hipk and Ems in an enzyme-substrate relationship. Given the strong conservation of Ems and Emx1/Emx2, it seems likely that a regulation of Emx1/Emx2 via phosphorylation by HIPK2 would also be conserved in vertebrates.

Finally, we could show that Muscle segment homeobox (Msh) is a direct target for phosphorylation by Hipk. Msh was a promising candidate for being an interaction partner of Hipk because it belongs to the NK-like factors, which are already known to interact with vertebrate HIPK2 [1]. In *Drosophila*, Msh plays a key role in neurogenesis and myogenesis [17,18]. Hipk might have several possible ways of controlling the function of Msh during neurogenic processes, as follows: (i) By direct phosphorylation at one of the sites mapped in this paper, (ii) indirectly by modulation of the *wg* or *hh* signals that activate *msh* expression [21,22], or (iii) by differential regulation via both of the afore mentioned processes. Lately, Msh was shown to act as a dorsal patterning factor during embryonic brain development by directly inducing Dap expression, affecting cell quiescence of neural stem cells [56]. As changes in the phosphorylation status of a transcription factor may modulate its activity [57], the transcription factor function of Msh might be altered via Hipk mediated phosphorylation and lead to altered gene expression and cell behavior.

The mammalian homologues MSX1–3 show some conformity with the expression of *msh* in *Drosophila* [58,59,60], so it might be interesting to investigate a potential conservation of Hipk/HIPK2 mediated phosphorylation. Similar to the situation in *Drosophila*, MSX genes are involved in the dorsal patterning of the vertebrate neural tube and the embryonal brain [61,62].

MSX genes are related to several forms of cancer. In accordance with their role during the development of craniofacial structures, for example, there are changes in the expression pattern of MSX genes in odontogenic tumors [63,64]. Increased levels of MSX2 in breast cancer samples were correlated with a good general prognosis [65]. MSX1 is described as a negative regulator of glioblastoma cell migration and invasion via inhibition of the Wnt/β-catenin signaling pathway [66]. A recent study also describes MSX2 as playing a crucial role in the progression of colorectal cancer [67]. HIPK2 itself is known to act as a tumor suppressor in β-catenin mediated skin tumorigenesis [68] and as a positive regulator of the pro-apoptotic tumor suppressor p53 [69,70]. It will be interesting to investigate if HIPK2 also exerts its influence on tumorigenic processes via the direct phosphorylation of MSX. Especially, a possible influence on the β-catenin mediated processes might shed light on the complex relationships between Wnt signaling and cancer. Altogether, this would add another dimension to the various levels of HIPK function and might possibly also offer new therapeutic approaches.

## 4. Materials and Methods

### 4.1. Bimolecular Fluorescence Complementation (BiFC)

All crosses were kept at 25 °C. Strains for BiFC analysis (pUAST-Hipk-Myc-NYFP, pUAST-NYFP-Myc-Hipk, and pUAST-Gro-HA-CYFP) were obtained as described [34]. pUAST-CYFP-HA-Hbn, pUAST-CYFP-HA-Ems, and pUAST-CYFP-HA-Msh were generated as follows. The full length coding sequences of *hbn*, *Ems, and Msh* were amplified from cDNA clones (*hbn*: cDNA, [30]; *ems*: clone cW13/7, [24]; *msh*: psC311, [71]) using the following primers: *hbn*: 5′-ATGATGACCACGACAACCTCG and 3′-TCAGTCCTCGCCCTTGGTG, *ems*: 5′-ATGACTAAGATGATTCCGCCG and 3′-TCAGTGGCTGGCGTCCAGCTCG, *msh*: 5′-ATGTTAAAGCTCAGCCCAGC and 3′-TTATCCCAGGTGCATCAGGCTC. PCR products were subcloned, sequence checked, and transferred by gateway cloning (Invitrogen, Carlsbad/California) into BiFC vectors [35]. The respective transgenes were generated via PhiC31-mediated integration into the landing site PBac{y+-attP-3B} (chromosome 3L, 65B2) [72,73]. A stock from Bloomington *Drosophila* Stock Centre was used as driver line, MS1096-Gal4 (BL 8860; w[1118] P{w[+mW.hs]=GawB}Bx[MS1096]). Immunostaining was used to enhance the YFP signal. Crosses of the driver line and single UAS-BiFC lines were used as technical controls (see Figure S1).

### 4.2. Purification of Recombinant Proteins

Constructs were generated using PCR, which was performed on *hbn* cDNA [30], *ems* cDNA (clone cW13/7, [24]), or *msh* cDNA (psC311, [71]) as a template, respectively. Primers came with unique restriction enzyme recognition sites added to their end, which enabled the direct cutting of PCR fragments. The next step was direct cloning from restricted, sticky-end PCR fragments into pGEX4T-1 (for *hbn*), pGEX5X-1 (for *ems*), or pGEX5X-2 (for *msh*) vector and transformation into bacteria. Some pGEX constructs were generated by restriction of existing pGEX constructs and reclosure by blunt end ligation (pGEX-MshC (from full length *msh* cDNA *Bam*HI/*Bgl*II); pGEX-MshNB-C1 (from pGEX-MshNB-C via *Sma*I/*Not*I), and pGEX-MshNB (from pGEX-MshNB-N2 via *Nae*I/*Not*I)). All constructs were confirmed by sequencing. Purification of the GST fusion proteins was performed via batch purification using Glutathione Sepharose^TM^ 4B (GE Healthcare, Frankfurt/Germany), according to the manufacturer’s instructions. The primer combinations we used are listed in Table S1.

### 4.3. In Vitro Mutagenesis

To introduce Ser/Ala exchanges in constructs, PCR mutagenesis was performed on cDNA (see above) as a template, and the mutation was introduced by a mutated primer. All constructs were sequence checked. Cloning and purification of the resulting constructs was processed as mentioned above.

### 4.4. In Vitro Phosphorylation Assay

To analyse the phosphorylation of recombinant proteins, in vitro kinase assays and visualization via autoradiography were performed with recombinant GST-Hipk (~8 µg per reaction), as described previously [33]. For mutational analysis, the used amounts of protein were individually adjusted to equal amounts for each group of wildtype protein and respective mutated forms. As a positive control, we used GST-tagged recombinant *Drosophila* Groucho (Gro) protein, which had previously been identified as a substrate of Hipk [3,33]. GST served as a negative control to exclude false positive signals by phosphorylation of the tag. We used Hipk without any substrate to illustrate the background caused by the kinase’s autophosphorylation activity.

### 4.5. Immunostaining

Preparation of imaginal discs and antibody staining was done according to standard protocols. Crawling third-instar larvae were dissected in phosphate-buffered saline (PBS) and fixed in freshly prepared phosphate lysine paraformaldehyde (2%) buffer for 1 h at room temperature. All washes were performed in PBX (PBS + 0.5% Triton X-100). Antibody incubation was done overnight at 4 °C after blocking with 5% normal horse serum at room temperature. The primary antibody used was anti-GFP (rabbit, 1:2000; Molecular Probes, A11122), the secondary antibody was Alexa Fluor 488-conjugated goat anti-rabbit (GARb, 1:1000; Molecular Probes, R37116). Stained imaginal discs were mounted in Vectashield H-1000 (Vector Laboratories, Burlingame, CA, USA). Images were obtained using confocal microscopy (Leica SP5; Leica, Wetzlar, Germany) and processed with IMAGEJ (NIH, Bethesda, MD, USA) and ADOBE ILLUSTRATOR CS6 (Adobe Systems, San Jose, CA, USA).

## 5. Conclusions

Here, we show that Hbn, Ems, and Msh interact with Hipk, as demonstrated by in vivo BiFC assays. The *Drosophila* homeodomain proteins Hbn, Ems, and Msh are in vitro substrates of Hipk. We mapped several phosphorylation sites of each protein. All newly identified substrates of Hipk fulfil functions during the development of the *Drosophila* nervous system. Furthermore, Hipk and the homeodomain proteins Hbn, Ems, and Msh have homologous vertebrate counterparts. Therefore, our results may be of general interest with respect to the role of HIPK/Hipk during neurogenesis, not only for *Drosophila*, but also for vertebrates.

## Supplementary Materials

Supplementary materials can be found at https://www.mdpi.com/1422-0067/20/8/1931/s1.

## Abbreviations

HIPK/Hipk	Homeodomain-interacting protein kinase
HD	Homeodomain
VNC	Ventral nerve cord
aa	amino acids
GFP	Green fluorescent protein
YFP	Yellow fluorescent protein
GST	Glutathion S-transferase
