# Supplementary material for: Drosophila Homeodomain-Interacting Protein Kinase (Hipk) Phosphorylates the Homeodomain Proteins Homeobrain, Empty Spiracles, and Muscle Segment Homeobox
Source: Int J Mol Sci. 2019 Apr 19;20(8):1931. doi: 10.3390/ijms20081931 (PMC6515119; doi:10.3390/ijms20081931)
Supplement: Supplementary file 1 [file ijms-20-01931-s001.zip › ijms-486784 - supplementary Figures.pdf]

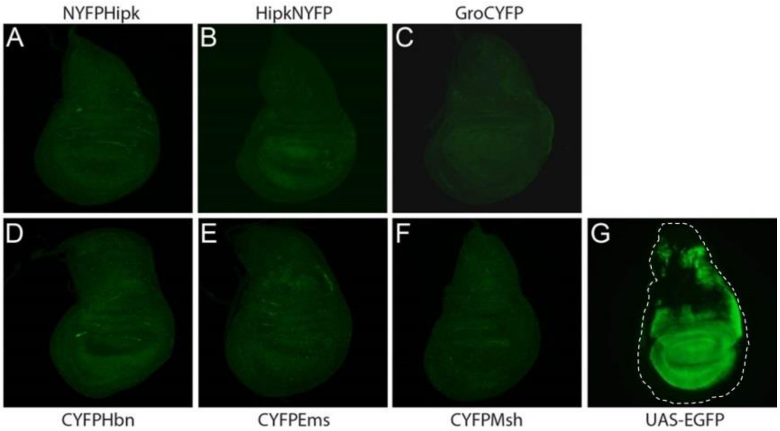

Figure S1. Expression of the single UAS-BiFC constructs driven by the MS1096-Gal4 driver. All discs, except for (**G**), were stained following the same protocol as done after co-expression of multiple UAS-BiFC constructs in Figure 2. **(A)** UASNYFP*hipk*, **(B)** UAS*hipk*NYFP, **(C)** UAS*gro*CYFP, **(D)** CYFP*hbn*, **(E)** UASCYFP*ems*, and **(F)** UASCYFP*msh*. Brightness of the images had to be artificially increased in order to properly visualize the discs, thereby leading to a slight baseline signal in some of the constructs. However, the signal strength is still clearly below the strength of the signals generated by complementation of the split YFP fragments owing to protein-protein interactions, making them clearly distinguishable. **(G)** MS1096 > UAS-enhanced green fluorescent protein (EGFP), illustrating the exact expression pattern of MS1096 driver spread over the pouch and hinge of the wing imaginal disc.

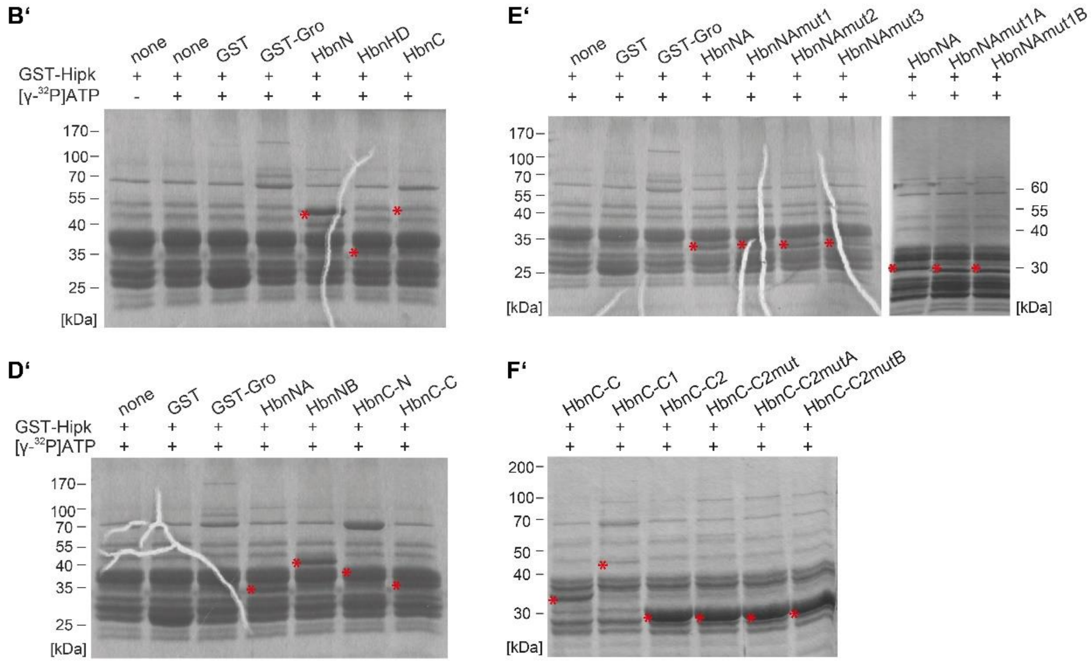

Figure S2. Original (Coomassie stained) protein gels/loading control referring to Figure 2. Sodium dodecyl sulfate-polyacrylamide gel electrophoresis (SDS-PAGE) corresponding to the in vitro phosphorylation assays analyzing the phosphorylation of glutathione S-transferase-Hbn constructs. The GST-Hipk protein (173 kDa) is subject to degradation, leading to numerous protein bands partially interfering with protein bands representing GST-Hbn. Marker used: **(B', E',** and **D')** Page Ruler Prestained Protein Ladder **(F')** Page Ruler Unstained (Thermo Fisher Scientific, Waltham, MA, USA). Red asterisks mark the expected size of the protein bands.

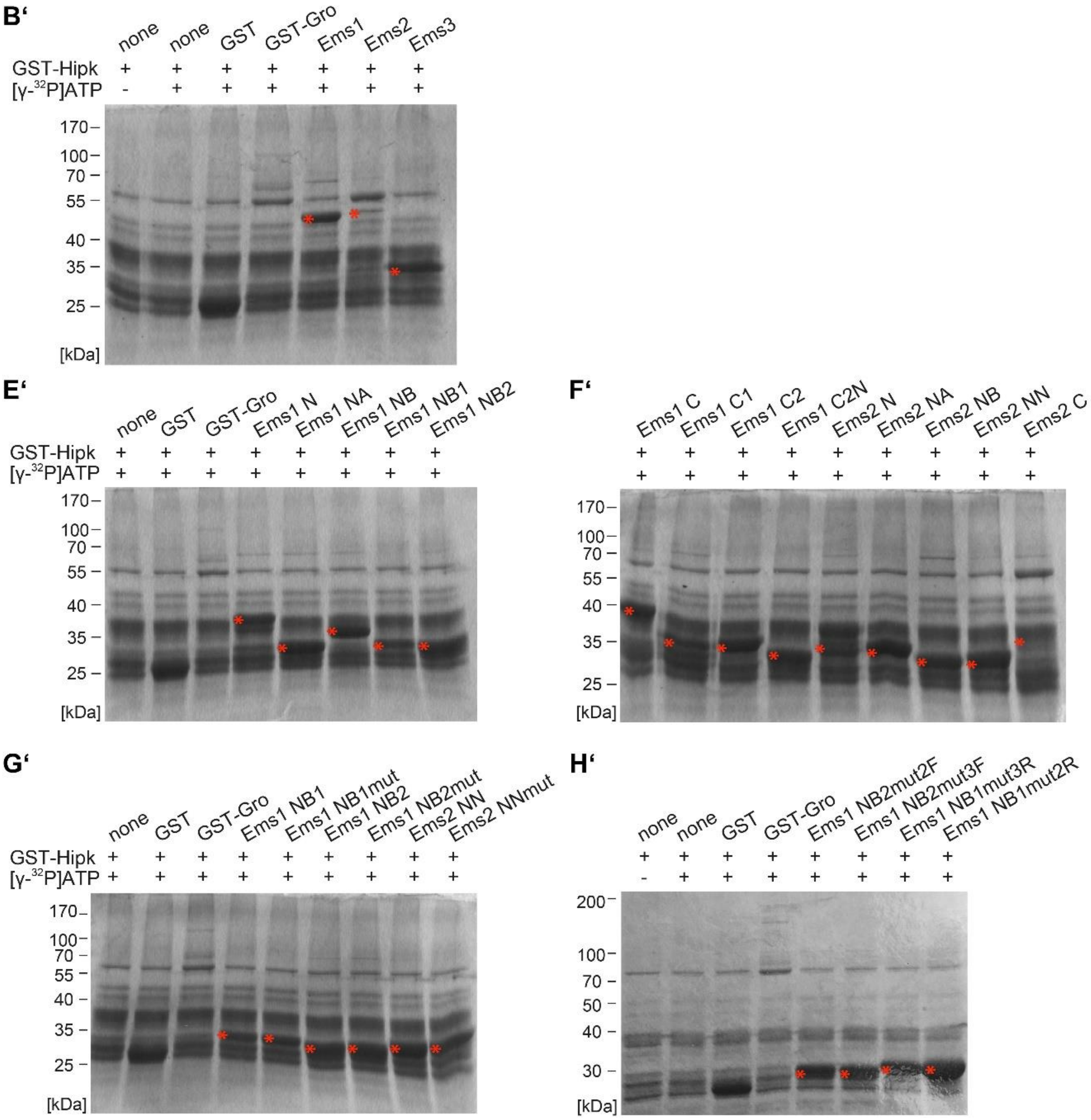

Figure S3. Original (Coomassie stained) protein gels/loading control referring to Figure 3. Sodium dodecyl sulfate-polyacrylamide gel electrophoresis (SDS-PAGE) corresponding to the in vitro phosphorylation assays analyzing the phosphorylation of glutathione S-transferase-Ems constructs. The GST-Hipk protein (173 kDa) is subject to degradation, leading to numerous protein bands partially interfering with protein bands representing GST-Ems. Marker used: **(B′, E′, F′,** and **G′)** Page Ruler Prestained Protein Ladder (**H′**) Page Ruler Unstained (Thermo Fisher Scientific, Waltham, MA, USA). Red asterisks mark the expected size of the protein bands.

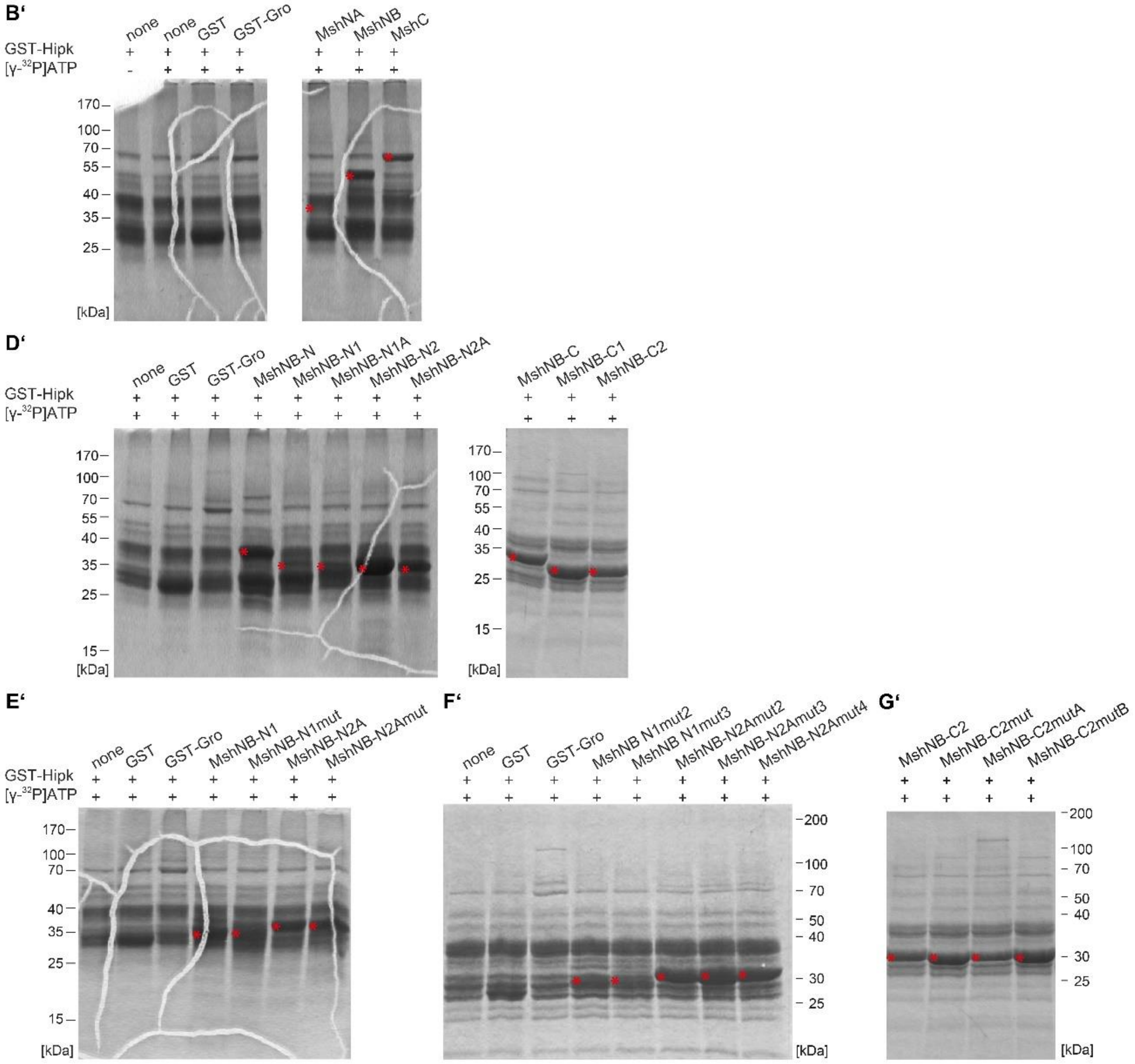

Figure S4. Original (Coomassie stained) protein gels/loading control referring to Figure 4. Sodium dodecyl sulfate-polyacrylamide gel electrophoresis (SDS-PAGE) corresponding to the *in vitro* phosphorylation assays analyzing the phosphorylation of glutathione S-transferase-Msh constructs. The GST-Hipk protein (173 kDa) is subject to degradation, leading to numerous protein bands partially interfering with protein bands representing GST-Msh. Marker used: (**B′, D′** and **E′**) Page Ruler Prestained Protein Ladder (**F′** and **G′**) Page Ruler Unstained (Thermo Fisher Scientific, Waltham, MA, USA). Red asterisks mark the expected size of the protein bands.
